# Supplementary material for: The PTM profiling of CTCF reveals the regulation of 3D chromatin structure by O-GlcNAcylation
Source: Nat Commun. 2024 Apr 1;15:2813. doi: 10.1038/s41467-024-47048-3 (PMC10985093; doi:10.1038/s41467-024-47048-3)
Supplement: Supplementary file 1 — Supplementary Information [file 41467_2024_47048_MOESM1_ESM.pdf]

The PTM profiling of CTCF reveals the regulation of 3D chromatin structure by O-GlcNAcylation

Xiuxiao Tang[1,2,3,4,5,14], Pengguihang Zeng[1,2,3,4,14], Kezhi Liu[1,2,3,4], Li Qing[1,2,3,4], Yifei Sun[1,2,3,4], Xinyi Liu[1,2,3,4], Lizi Lu[1,2,3,4], Chao Wei[1,2,3,4], Jia Wang[6], Shaoshuai Jiang[1,2,3,4], Jun Sun[7,8], Wakam Chang[9], Haopeng Yu[7,8], Hebing Chen[10], Jiaguo Zhou[5], Chengfang Xu[11,*], Lili Fan[12,*], Yi-Liang Miao[13,*], Junjun Ding[1,2,3,4,7,*]

[1] RNA Biomedical Institute, Sun Yat-Sen Memorial Hospital, Zhongshan School of Medicine, Sun Yat-Sen University, Guangzhou 510080, China

[2] Department of Rehabilitation Medicine, The Seventh Affiliated Hospital, Sun Yat-Sen University, Shenzhen, Guangdong, 518107, China

[3] Advanced Medical Technology Center, The First Affiliated Hospital, Zhongshan School of Medicine, Sun Yat-Sen University, Guangzhou, Guangdong, China

[4] Center for Stem Cell Biology and Tissue Engineering, Key Laboratory for Stem Cells and Tissue Engineering, Ministry of Education, Sun Yat-Sen University, Guangzhou 510080, China

[5] Department of Pharmacology and Cardiac & Cerebral Vascular Research Center, Zhongshan School of Medicine, Sun Yat-Sen University, Guangzhou 510080, China

[6] GMU-GIBH Joint School of Life Sciences, Guangzhou Medical University, Guangzhou, 511436, China

[7] West China Biomedical Big Data Center, West China Hospital/West China School of Medicine, Sichuan University, Chengdu 610041, CHINA

[8] Med-X Center for Informatics, Sichuan University, Chengdu 610041, China

[9] Department of Biomedical Sciences, Faculty of Health Sciences, University of Macau, Taipa, Macau, China

[10] Institute of Health Service and Transfusion Medicine, Beijing 100850, China

[11] The obstetric and gynecology Department of The third affiliated hospital of Sun Yat-Sen University

[12] Guangzhou Key Laboratory of Formula-Pattern of Traditional Chinese Medicine, School of Traditional Chinese Medicine, Jinan University, Guangzhou, Guangdong, China

[13] Institute of Stem Cell and Regenerative Biology, College of Animal Science and Veterinary Medicine, Huazhong Agricultural University, Wuhan, Hubei, China

[14] These authors contributed equally to the work

* Correspondence: xuchengf@mail.sysu.edu.cn; fanlili@jnu.edu.cn; miaoyl@mail.hzau.edu.cn; dingjunj@mail.sysu.edu.cn

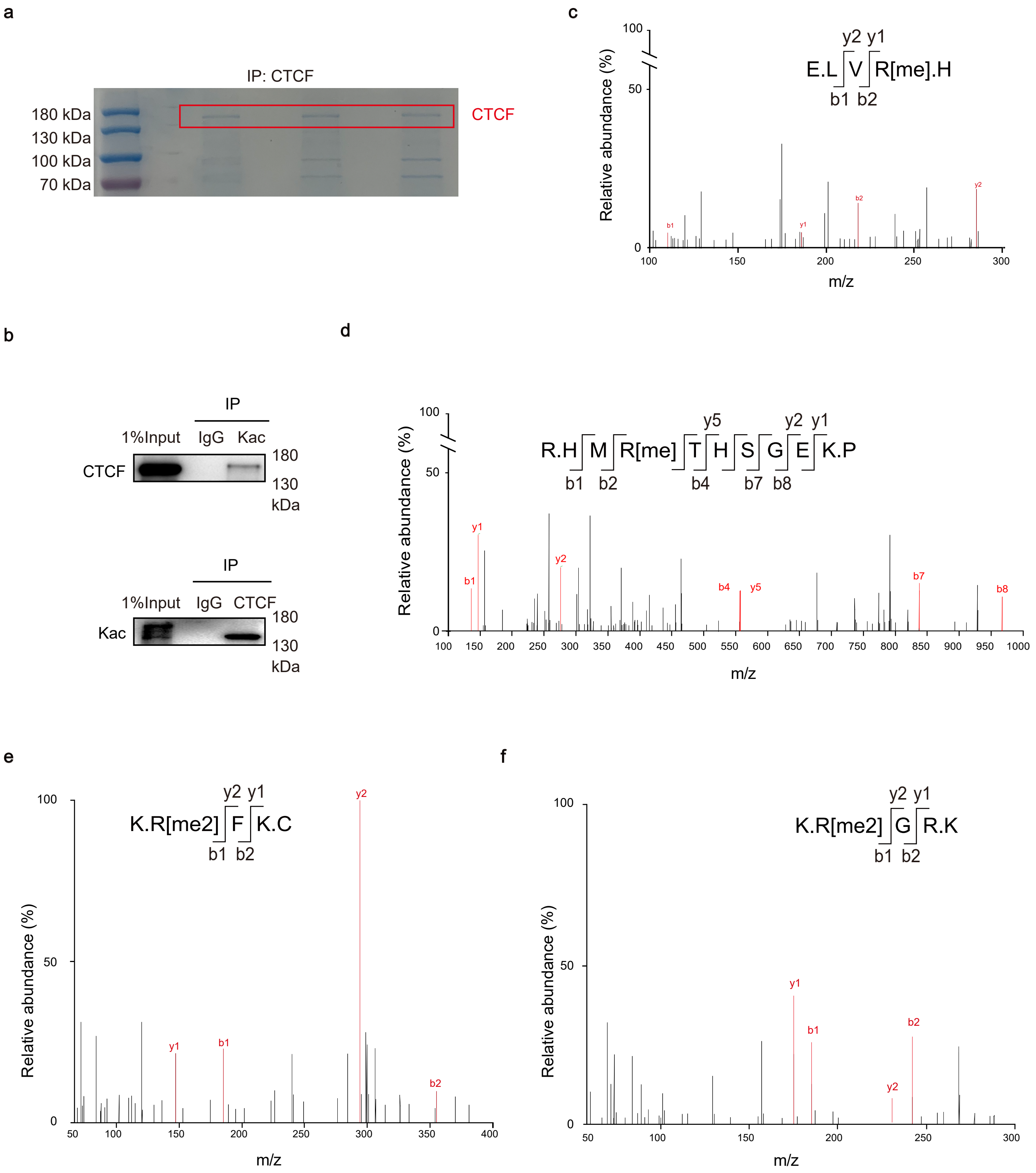
a
IP: CTCF
180 kDa
130 kDa
100 kDa
70 kDa
CTCF
b
IP
1%Input
IgG
Kac
CTCF
180
130
kDa
IP
1%Input
IgG
CTCF
Kac
180
130
kDa
c
Relative abundance (%)
m/z
y2 y1
E.L V R[me].H
b1 b2
d
Relative abundance (%)
m/z
y5 y2 y1
R.H M R[me] T H S G E K.P
b1 b2 b4 b7 b8
e
Relative abundance (%)
m/z
y2 y1
K.R[me2] F K.C
b1 b2
f
Relative abundance (%)
m/z
y2 y1
K.R[me2] G R.K
b1 b2

Supplementary Fig. 1 The purification of CTCF, Related to Fig. 1. a, Coomassie blue staining for purification of CTCF. This experiment was performed three times, with similar results. b, Whole-cell lysates were denatured and incubated with Kac-conjugated agarose beads to enrich acetylated proteins and bound proteins were analyzed by western blot (top) and whole-cell lysates were denatured and immunoprecipitated with antibodies for CTCF. Acetylation of proteins was detected by western blot with Kac (bottom). This experiment was performed three times, with similar results. c-f, LC-MS/MS spectra of methylated peptides of endogenous CTCF derived from mESCs and CTCF was purified by immunoprecipitation with antibody. The matched fragment ions were labeled in red. Source data are provided as a Source Data file.

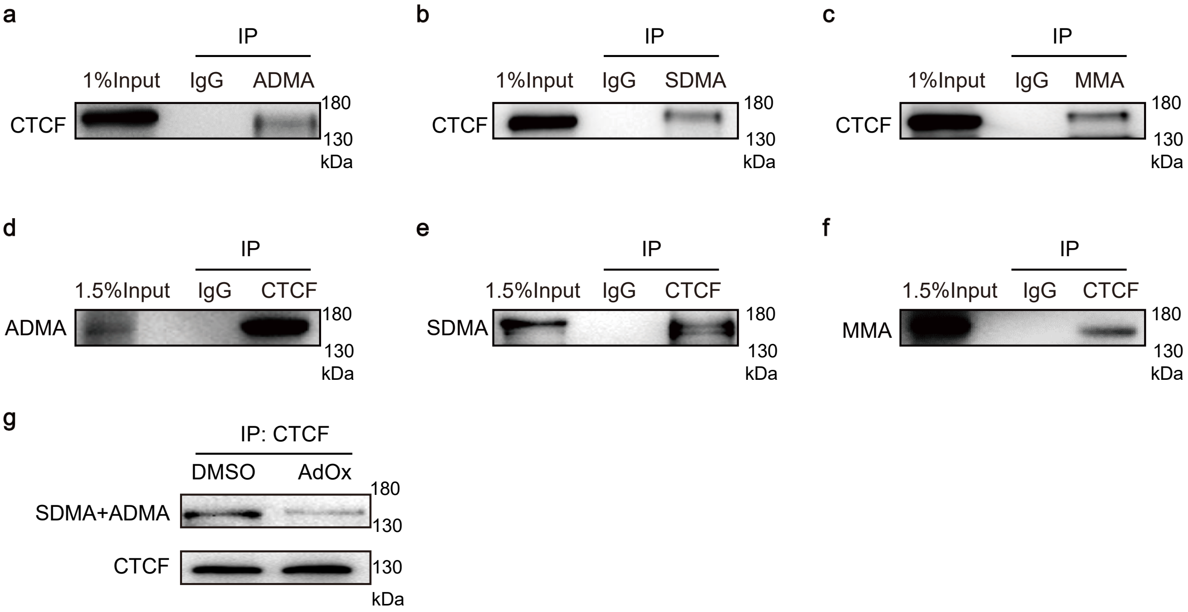

Supplementary Fig. 2 Validation of arginine methylation, Related to Fig. 2. a-c, Whole-cell lysates were denatured and incubated with ADMA (a), SDMA (b) and MMA (c) -conjugated agarose beads to enrich methylated proteins respectively, and bound proteins were analyzed by western blot. This experiment was performed three times, with similar results. d-f, Whole-cell lysates were denatured and immunoprecipitated with antibody for CTCF. Methylation of proteins was detected by western blot with ADMA (d), SDMA (e) and MMA (f), respectively. This experiment was performed three times, with similar results. g, The methylation signal of CTCF decreased when treated with an arginine methylation inhibitor, periodate oxidized adenosine (AdOx). This experiment was performed three times, with similar results. ADMA: asymmetrical dimethylarginine; SDMA: symmetrical dimethylarginine; MMA: mono-methylarginine. Source data are provided as a Source Data file.

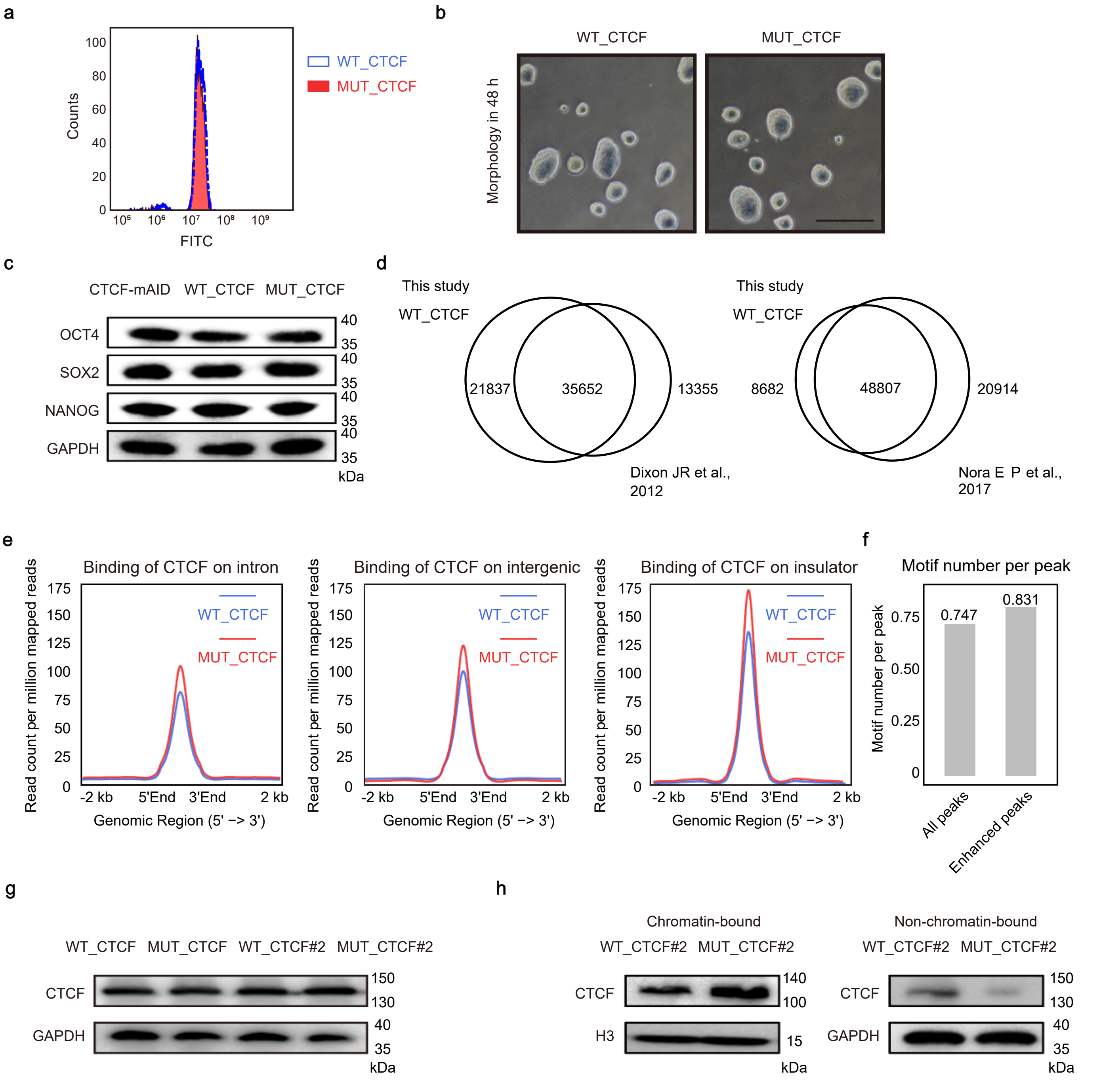

a
100
80
60
40
20
0
Counts
FITC
WT_CTCF
MUT_CTCF
b
WT_CTCF
MUT_CTCF
Morphology in 48 h
c
CTCF-mAID WT_CTCF MUT_CTCF
OCT4
SOX2
NANOG
GAPDH
40
35
kDa
d
This study
WT_CTCF
21837
35652
13355
Dixon JR et al., 2012
8682
48807
20914
Nora E P et al., 2017
e
Binding of CTCF on intron
Binding of CTCF on intergenic
Binding of CTCF on insulator
Read count per million mapped reads
175
150
125
100
75
50
25
0
-2 kb 5'End 3'End 2 kb
Genomic Region (5' –> 3')
f
Motif number per peak
0.747
0.831
Motif number per peak
0.75
0.50
0.25
0
All peaks
Enhanced peaks
g
WT_CTCF MUT_CTCF WT_CTCF#2 MUT_CTCF#2
CTCF
GAPDH
150
130
40
35
kDa
h
Chromatin-bound
Non-chromatin-bound
WT_CTCF#2 MUT_CTCF#2
CTCF
H3
GAPDH
140
100
15
150
130
40
35
kDa

Supplementary Fig. 3 Validation of cell lines and bioinformatics analysis of CTCF ChIP-seq, Related to Fig. 3. a, FACS analysis showing the consistent expression of CTCF in WT_CTCF and MUT_CTCF mESCs. b, The cell morphology of WT_CTCF and MUT_CTCF mESCs in 48 h. Scale bar was 500 μm. c, Western blot of total cell lysates from CTCF-mAID mESCs, WT_CTCF cells and MUT_CTCF cells to detect the expression of OCT4, SOX2 and NANOG after 48 h auxin treatment. GADPH was shown as a loading control. d, Venn diagram showing the overlap between CTCF ChIP-seq peaks of WT_CTCF mESCs in this study and two published CTCF ChIP-seq datasets. e, Profiling of CTCF binding indicating the overall binding of O-GlcNAc-deficient CTCF on intron (left), intergenic (middle) and insulator (right) were enhanced. f, Boxplot showing there were more CTCF motifs on the enhanced peaks. g, Western blot of total cell lysates from WT_CTCF cells, MUT_CTCF cells, WT_CTCF clone#2 and MUT_CTCF clone#2 to detect the expression of CTCF. GAPDH was showing as a loading control. h, Western blot of chromatin-bound CTCF (left) and non-chromatin-bound CTCF (right) in WT_CTCF clone#2 and MUT_CTCF clone#2. H3 and GAPDH were showing as loading control. All western blot experiments were performed three times, with similar results. Source data are provided as a Source Data file.

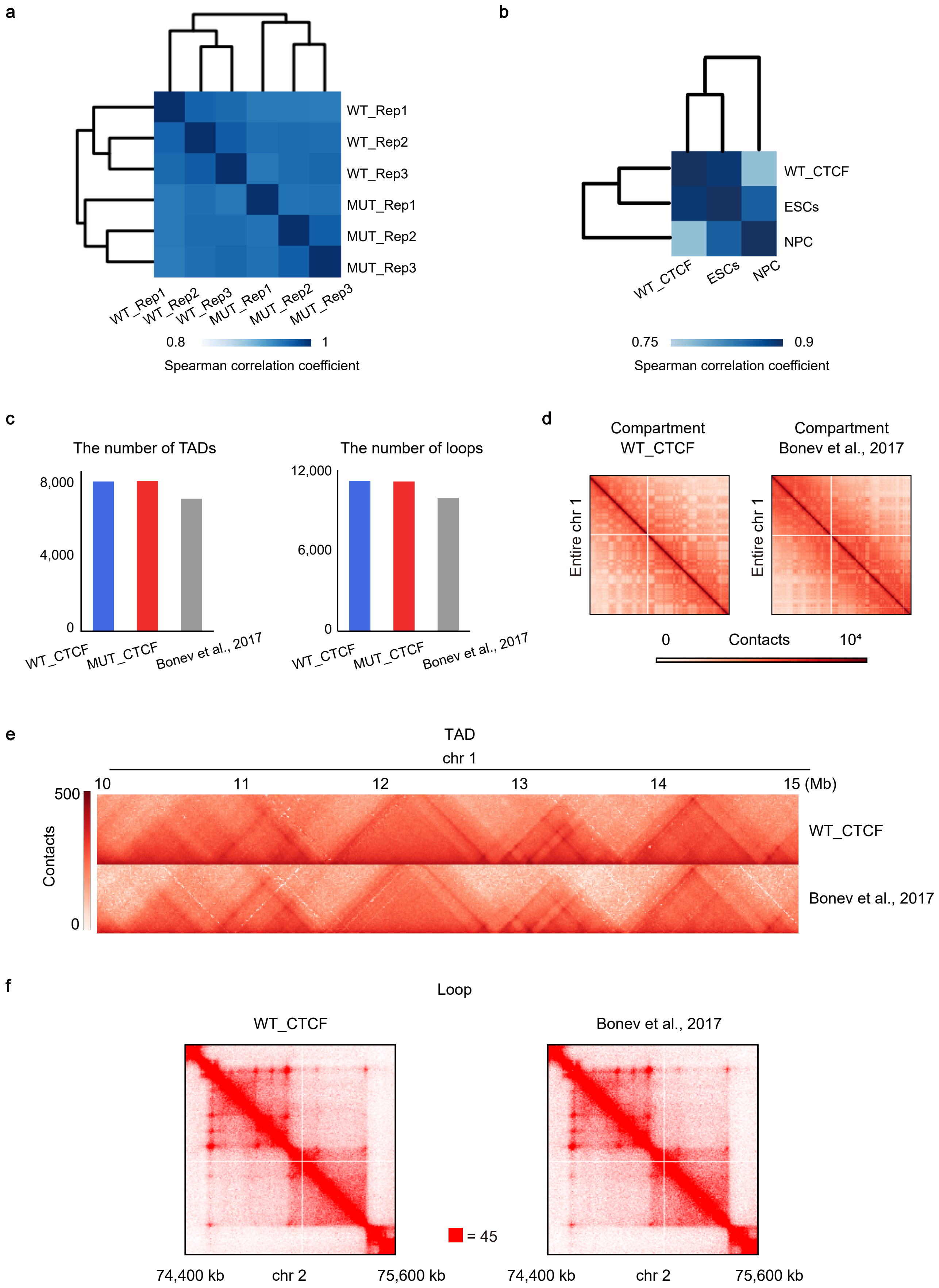

a
WT_Rep1
WT_Rep2
WT_Rep3
MUT_Rep1
MUT_Rep2
MUT_Rep3
0.8
1
Spearman correlation coefficient
b
WT_CTCF
ESCs
NPC
0.75
0.9
Spearman correlation coefficient
c
The number of TADs
8,000
4,000
0
The number of loops
12,000
6,000
0
WT_CTCF
MUT_CTCF
Bonev et al., 2017
d
Compartment WT_CTCF
Compartment Bonev et al., 2017
Entire chr 1
0
Contacts
10⁴
e
TAD
chr 1
10
11
12
13
14
15 (Mb)
500
Contacts
0
WT_CTCF
Bonev et al., 2017
f
Loop
WT_CTCF
Bonev et al., 2017
= 45
74,400 kb
chr 2
75,600 kb

Supplementary Fig. 4 Quality control metrics of Hi-C data, Related to Fig 4. a, Spearman correlation coefficients among and between the WT_CTCF and MUT_CTCF conditions. b, Spearman correlation coefficients between the WT_CTCF in this study, ESCs and neural progenitor cells (NPCs) in Bonev et al., 2017. c, The number of TADs and loops identified in this study and in Bonev et al., 2017. d-f, Hi-C contact heatmaps showing compartments (d), TADs (e), loops (f) in this study and in Bonev et al., 2017. Source data are provided as a Source Data file.

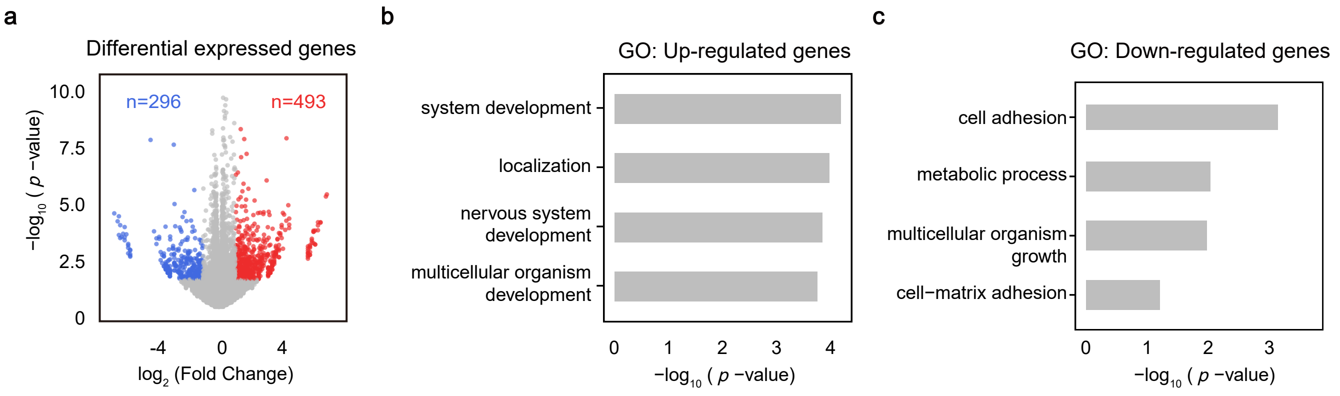

Supplementary Fig. 5 Deficiency of CTCF O-GlcNAcylation affects gene expression, Related to Fig 6. a, Volcano plot comparing gene expression in MUT_CTCF mESCs against WT_CTCF mESCs. Gray dots, genes with no significant change; blue dots, down-regulated genes; red dots, up-regulated genes, p-values by exact test based on over-dispersed Poisson regression, performed by R package edgeR. b, GO analysis revealed the up-regulated genes were associated with developmental processes, p-values by Fisher's Exact test. c, GO analysis revealed the down-regulated genes were associated with cell adhesion and metabolic process, p-values by Fisher's Exact test. Source data are provided as a Source Data file.

a

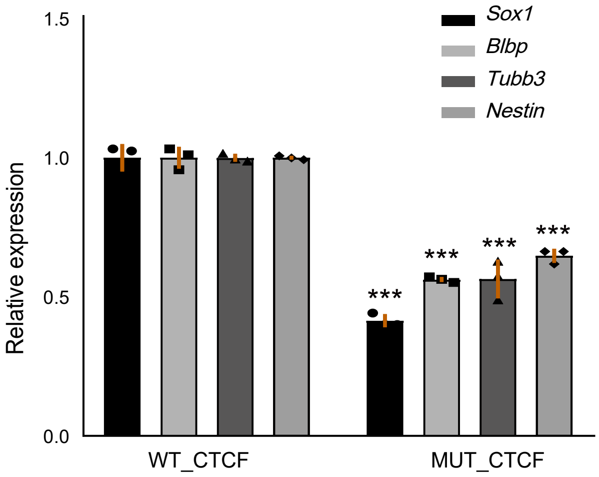

Supplementary Fig. 6 Mutation of CTCF O-GlcNAcylation inhibits the differentiation into NPCs, Related to Fig 7. a, Quantitative PCR analysis of NPCs markers. All statistical tests were two-sided Student t-test. Significance levels were indicated by asterisks: **, p < 0.01; ***, p < 0.001. All data were presented as mean ± SD (n = 3 biologically independent samples). Source data are provided as a Source Data file.

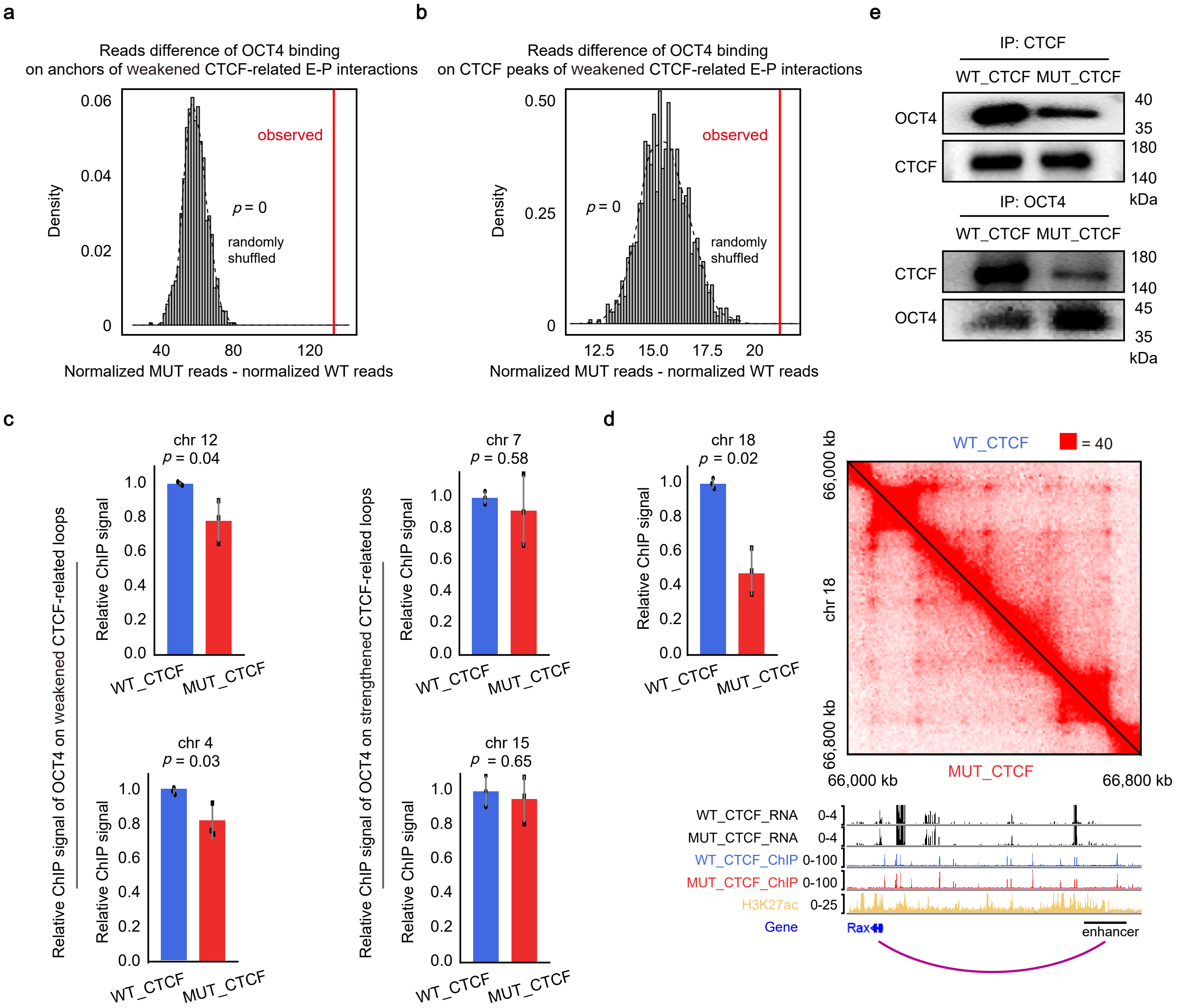
a
Reads difference of OCT4 binding
on anchors of weakened CTCF-related E-P interactions
observed
p = 0
randomly shuffled
Density
Normalized MUT reads - normalized WT reads
b
Reads difference of OCT4 binding
on CTCF peaks of weakened CTCF-related E-P interactions
observed
p = 0
randomly shuffled
Density
Normalized MUT reads - normalized WT reads
e
IP: CTCF
WT_CTCF MUT_CTCF
OCT4
CTCF
IP: OCT4
WT_CTCF MUT_CTCF
CTCF
OCT4
kDa
c
Relative ChIP signal of OCT4 on weakened CTCF-related loops
chr 12 p = 0.04
chr 4 p = 0.03
Relative ChIP signal of OCT4 on strengthened CTCF-related loops
chr 7 p = 0.58
chr 15 p = 0.65
Relative ChIP signal
WT_CTCF
MUT_CTCF
d
chr 18 p = 0.02
WT_CTCF
= 40
MUT_CTCF
66,000 kb
66,800 kb
WT_CTCF_RNA 0-4
MUT_CTCF_RNA 0-4
WT_CTCF_ChIP 0-100
MUT_CTCF_ChIP 0-100
H3K27ac 0-25
Gene
Rax
enhancer

Supplementary Fig. 7 Weakened CTCF-related E-P interactions exhibit a higher level of OCT4 signals. a, Permutation test showing OCT4 had higher level of binding on anchors of weakened CTCF-related E-P interactions than randomly shuffled set of loops. p-value by one-tailed test based on the assumption of normal distribution, p=0. b, Permutation test showing OCT4 had higher level of binding on CTCF peaks of weakened CTCF-related E-P interactions than randomly shuffled set of loops. p-value by one-tailed test based on the assumption of normal distribution, p=0. c, ChIP-qPCR showing the enrichment of OCT4 on the anchors of weakened (left) or strengthened (right) CTCF-related loops. All statistical tests were two-sided Student t-test. All data were presented as mean ± SD (n = 3 biologically independent samples). d, Representative genomic loci showing weakened loop targeted genes and ChIP-qPCR showing the enrichment of OCT4 on the anchors of weakened loops. Data were presented as mean ± SD (n = 3 biologically independent samples). e, Western blot showing that mutation of CTCF O-GlcNAcylation weakened the interaction between CTCF and OCT4. Source data are provided as a Source Data file.

a

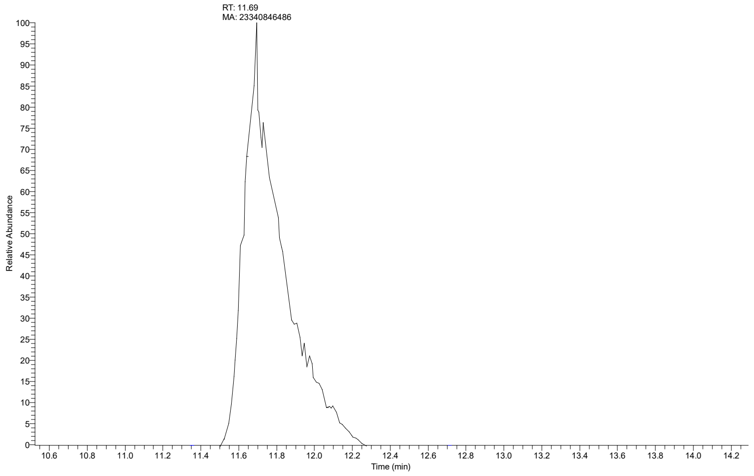

b

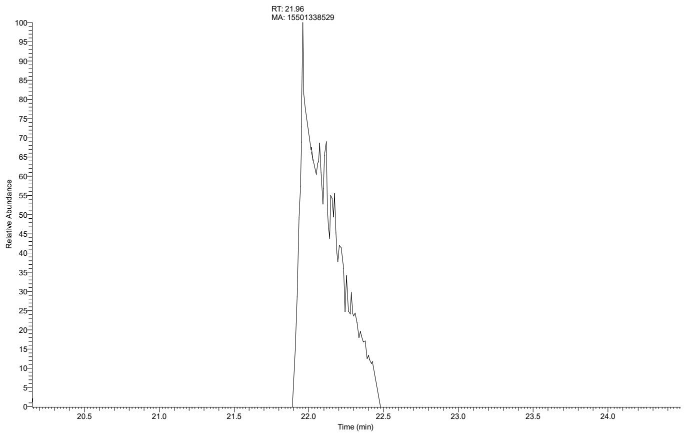

Supplementary Fig. 8. Extracted ion chromatograms of peptide. a, Extracted ion chromatograms of the O-GlcNAcylated peptide. b, Extracted ion chromatograms of the unmodified peptide.

Supplementary Table 1. ChIP-seq statistics

Sample	Total	Unique	Multiple	Unmapped
WT_CTCF.R1	74480336	53126196	15070262	6283878
WT_CTCF.R2	67958130	48348223	13757748	5852159
MUT_CTCF.R1	71157312	49830929	17666060	3660323
MUT_CTCF.R2	73646740	51575000	18231272	3840468
WT_CTCF_input.R1	63120404	38638698	20748354	3733352
MUT_CTCF_input.R1	57369566	21053135	31171469	5144962

Supplementary Table 2. Oligonucleotides

Primers used in this paper	
Fgf5 realtime-F	TGTGTCTCAGGGGATTGTAGG
Fgf5 realtime-R	AGCTGTTTTCTTGGAATCTCTCC
Pax3 realtime-F	GCAGCGCAGGAGCAGAACCA
Pax3 realtime-R	GCACTCGGGCCTCGGTAAGC
Mixl1 realtime-F	ATCCGCCCGGACCCTCCAAA
Mixl1 realtime-R	TCGGTTCTGGAACCACACCTGGA
Gata4 realtime-F	CCCTACCCAGCCTACATGG
Gata4 realtime-R	ACATATCGAGATTGGGGTGTCT
Gata6 realtime-F	TTGCTCCGGTAACAGCAGTG
Gata6 realtime-R	GTGGTCGCTTGTGTAGAAGGA
Cdx2 realtime-F	CAAGGACGTGAGCATGTATCC
Cdx2 realtime-R	GTAACCACCGTAGTCCGGGTA
Oct3/4 realtime-F	GTGGAGGAAGCCGACAACAATGA
Oct3/4 realtime-R	CAAGCTGATTGGCGATGTGAG
Nanog realtime-F	TGGTCCCCACAGTTTGCCTAGTTC
Nanog realtime-R	CAGGTCTTCAGAGGAAGGGCGA
Sox2 realtime 1-F	CTGGACTGCGAACTGGAGAAG
Sox2 realtime 1-R	AATTTGGATGGGATTGGTGGT
sox1 realtime-F	GTGACATCTGCCCCCATC
sox1 realtime-R	GAGGCCAGTCTGGTGTCAG
Nestin realtime-F	CTGCAGGCCACTGAAAAGTT
Nestin realtime-R	TTCAGCCAAGGAGACAGTCA
Blbp realtime-F	GGCAAGATGGTCGTGACTCT
Blbp realtime-R	ACCTCCACACCGAAGACAAA
Tubb3 realtime-F	AACCAGATAGGGGCCAAGTT
Tubb3 realtime-R	GGCCTGAATAGGTGTCCAAA

Supplementary Table 3. RNA-seq statistics

Sample	Total	Unique	Multiple
MUT.RNA.R1	25141990	23201868	1192166
MUT.RNA.R2	24755332	22870517	1142240
WT.RNA.R1	25185745	23211826	1197890
WT.RNA.R2	25253995	23210290	1197976

Supplementary Table 4 The quality control steps of Hi-C

Supplementary Table 4-1 Step 1 of Hi-C data quality control

Sample Name	Total Pairs	Reported Pairs	Reported Ratio
WT_Rep1	891882490	532314732	59.68%
WT_Rep2	1541879172	887438717	57.56%
WT_Rep3	1698451224	1034842720	60.93%
MUT_Rep1	960728847	560114338	58.30%
MUT_Rep2	1318284107	747740724	56.72%
MUT_Rep3	1500762042	888468710	59.20%

Reads were aligned to mm9 reference genome using bowtie2, reads with mapping quality > 10 were assigned to MboI restriction fragments and interaction pairs were reconstructed as total pairs. Singleton or multi-hits pairs are filtered out, remaining pairs were denoted as reported pairs.

Supplementary Table 4-2. Step 2 of Hi-C data quality control

Sample Name	Valid Interaction Pairs	FF	RR	RF	FR	Ligation Efficiency
WT_Rep1	508314911	126956223	126873742	123851812	130633134	95.49%
WT_Rep2	846400045	211389337	211263652	206277665	217469391	95.38%
WT_Rep3	991209046	247498126	247423168	241458757	254828995	95.78%
MUT_Rep1	527405953	131857704	131755235	128608628	135184386	94.16%
MUT_Rep2	704704231	176138645	176029929	171798445	180737212	94.24%
MUT_Rep3	839842049	209886003	209778087	204703111	215474848	94.53%

Among reported pairs, failed ligation products (dangling end pairs, re-ligation pairs, self-cycle pairs) and pairs not able to reconstruct the ligation product were discarded, remaining pairs were denoted as valid interaction pairs. Valid interaction pairs were divided into 4 classes by the fragment directions (F for forward, R for reverse). As the ligation is a random process, about 25% of each valid ligation class is expected.

Supplementary Table 4-3. Step 3 of Hi-C data quality control

Sample Name	Valid RMDUP Pairs	PCR Duplication Ratio	Long Intra Interaction	Long Intra Ratio	Library Efficiency
WT_Rep1	490790030	3.45%	313371562	63.85%	55.03%
WT_Rep2	798693864	5.64%	509567229	63.80%	51.80%
WT_Rep3	932545214	5.92%	595005195	63.80%	54.91%

MUT_Rep1	501713985	4.87%	289967099	57.80%	52.22%
MUT_Rep2	668322376	5.16%	386579144	57.84%	50.70%
MUT_Rep3	792218069	5.67%	458197529	57.84%	52.79%

Among valid interaction pairs, PCR duplicates were discarded, remaining pairs were denoted as valid remove duplication interaction pairs and used for further analysis. The fractions of intra and inter-chromosomal interactions as well as long range (>20kb) versus short range (<20kb) intra-chromosomal interactions were calculated. Finally, the overall library efficiency was calculated as (valid remove duplication interaction pairs) / (total pairs).
