# Supplementary material for: The PTM profiling of CTCF reveals the regulation of 3D chromatin structure by O-GlcNAcylation
Source: Nat Commun. 2024 Apr 1;15:2813. doi: 10.1038/s41467-024-47048-3 (PMC10985093; doi:10.1038/s41467-024-47048-3)
Supplement: Supplementary file 3 — Reporting Summary [file 41467_2024_47048_MOESM3_ESM.pdf]

nature portfolio

Corresponding author(s): Junjun Ding
Last updated by author(s): Mar 14, 2024

Reporting Summary

Nature Portfolio wishes to improve the reproducibility of the work that we publish. This form provides structure for consistency and transparency in reporting. For further information on Nature Portfolio policies, see our Editorial Policies and the Editorial Policy Checklist.

Statistics

For all statistical analyses, confirm that the following items are present in the figure legend, table legend, main text, or Methods section.

n/a	Confirmed	
☐	☒	The exact sample size (*n*) for each experimental group/condition, given as a discrete number and unit of measurement
☐	☒	A statement on whether measurements were taken from distinct samples or whether the same sample was measured repeatedly
☐	☒	The statistical test(s) used AND whether they are one- or two-sided<br>*Only common tests should be described solely by name; describe more complex techniques in the Methods section.*
☒	☐	A description of all covariates tested
☒	☐	A description of any assumptions or corrections, such as tests of normality and adjustment for multiple comparisons
☐	☒	A full description of the statistical parameters including central tendency (e.g. means) or other basic estimates (e.g. regression coefficient) AND variation (e.g. standard deviation) or associated estimates of uncertainty (e.g. confidence intervals)
☐	☒	For null hypothesis testing, the test statistic (e.g. *F*, *t*, *r*) with confidence intervals, effect sizes, degrees of freedom and *P* value noted<br>*Give P values as exact values whenever suitable.*
☒	☐	For Bayesian analysis, information on the choice of priors and Markov chain Monte Carlo settings
☒	☐	For hierarchical and complex designs, identification of the appropriate level for tests and full reporting of outcomes
☐	☒	Estimates of effect sizes (e.g. Cohen's *d*, Pearson's *r*), indicating how they were calculated

Our web collection on statistics for biologists contains articles on many of the points above.

Software and code

Policy information about availability of computer code

Data collection	No software was used for data collection.
Data analysis	There are details in the Methods.<br>Trim Galore v0.4.4_dev<br>Bowtie2 v2.3.4.1<br>STAR_v2.5.4b<br>SAMtools v0.1.19-96b5f2294a<br>BEDTools v2.29.2<br>macs2 v2.1.1.20160309<br>ngsplot<br>deepTools v3.1.1<br>HTSeq-count v0.9.1<br>edgeR v3.246.3<br>HiC-Pro v2.10.0<br>Juicebox v7.0<br>CscoreTool<br>TopDom v0.0.2<br>diffloop v1.22.0<br>GENOVA v1.0.0<br>HICExplorer v3.4.1

ggplot2
PyMol 3.0

For manuscripts utilizing custom algorithms or software that are central to the research but not yet described in published literature, software must be made available to editors and reviewers. We strongly encourage code deposition in a community repository (e.g. GitHub). See the Nature Portfolio guidelines for submitting code & software for further information.

Data

Policy information about availability of data

All manuscripts must include a data availability statement. This statement should provide the following information, where applicable:

- Accession codes, unique identifiers, or web links for publicly available datasets
- A description of any restrictions on data availability
- For clinical datasets or third party data, please ensure that the statement adheres to our policy

Previously published raw reads of ChIP-seq data were downloaded from GSE29218 (CTCF), GSE85185 (CTCF), GSM2417096 (H3K27ac) and re-processed as described in methods. Previously published raw reads of Hi-C data were downloaded from GSE96107, and re-processed as described in methods. All datasets are available in GEO under the accession number GSE255897. Other information needed is available form corresponding author upon request. Source data are provided with this paper.

Research involving human participants, their data, or biological material

Policy information about studies with human participants or human data. See also policy information about sex, gender (identity/presentation), and sexual orientation and race, ethnicity and racism.

Reporting on sex and gender	N/A
Reporting on race, ethnicity, or other socially relevant groupings	N/A
Population characteristics	N/A
Recruitment	N/A
Ethics oversight	N/A

Note that full information on the approval of the study protocol must also be provided in the manuscript.

Field-specific reporting

Please select the one below that is the best fit for your research. If you are not sure, read the appropriate sections before making your selection.

☒ Life sciences ☐ Behavioural & social sciences ☐ Ecological, evolutionary & environmental sciences

For a reference copy of the document with all sections, see nature.com/documents/nr-reporting-summary-flat.pdf

Life sciences study design

All studies must disclose on these points even when the disclosure is negative.

Sample size	No statistical methods were chosen to determine sample size. Experiments are performed on cell line, with replicates (sample size) = 3 in Hi-C, ChIP-qPCR, cell viability assay, colony formation assay, immunofluorescence and western blotting; replicates (sample size) = 2 in RNA-seq, ChIP-seq and ATAC-seq. Sample sizes were chosen to provide sufficient material for experiment and statistical tests within affordable costs.
Data exclusions	No data were excluded from analyses.
Replication	Two biological replicates were performed for RNA-seq, ChIP-seq and ATAC-seq. For the functional experiment section, at least three times were performed. All attempts at replication were successful.
Randomization	In our study, we utilized a uniformly treated cell line for the experiments. Both the experimental and control groups were derived from this consistent condition, ensuring similarity in their baseline states. Given this uniformity and the controlled experimental environment, the processes of randomization or controlling for covariates are not relevant to our study.
Blinding	The investigators were not blinded to the group as no human subjects were involved and no subjective measurements were taken.

Reporting for specific materials, systems and methods

We require information from authors about some types of materials, experimental systems and methods used in many studies. Here, indicate whether each material, system or method listed is relevant to your study. If you are not sure if a list item applies to your research, read the appropriate section before selecting a response.

Materials & experimental systems

n/a	Involved in the study
☐	☒ Antibodies
☐	☒ Eukaryotic cell lines
☒	☐ Palaeontology and archaeology
☒	☐ Animals and other organisms
☒	☐ Clinical data
☒	☐ Dual use research of concern
☒	☐ Plants

Methods

n/a	Involved in the study
☐	☒ ChIP-seq
☐	☒ Flow cytometry
☒	☐ MRI-based neuroimaging

Antibodies

Antibodies used	Antibodies used in this study were anti-CTCF (Active Motif, # 61311), anti-CTCF (abclonal, #A19588), anti-RL2 (abcam, #ab2739), anti-OGT (GeneTex, # GTX109939), anti-Mono-Methyl Arginine (MMA) antibody (CST, #8015), anti-Symmetric Di-Methyl Arginine (SDMA) antibody (PTMBIO, #PTM-617RM), anti-Asymmetric Di-Methyl Arginine (ADMA) antibody (PTMBIO, #PTM-605RM) , anti-Acetyllysine antibody (PTMBIO, #PTM-105RM), anti-GADPH (abclonal, #A8419), anti-OCT4 (Santa, #sc-5279), anti-SOX2 (Santa, #sc-36582-3), anti-NANOG (Bethyl, #A300-397A), anti-H3(Santa, # sc-17576). Goat anti-rabbit-IgG (H+L)–HRP (CST, #7074s) and goat anti-mouse-IgG (H+L)-HRP (CST, #7076s) were used as secondary antibodies for western blotting.
Validation	Knockout validated

Eukaryotic cell lines

Policy information about cell lines and Sex and Gender in Research

Cell line source(s)	The mESCs lines used for this study were R1 (a kind gift from Dr. Shaorong Gao, PMID: 33357405). CTCF-EGFP-AID ESCs were presented by Bruneau lab (PMID:28525758)
Authentication	Cells lines are confirmed by a typical round shape ESCs morphology with small and tightly packed cells, and a high nucleus-to-cytoplasm ratio.
Mycoplasma contamination	Cell lines are negative for Mycoplasma contamination.
Commonly misidentified lines (See ICLAC register)	No commonly misidentified cell lines were used.

Plants

Seed stocks	N/A
Novel plant genotypes	N/A
Authentication	N/A

ChIP-seq

Data deposition

☒ Confirm that both raw and final processed data have been deposited in a public database such as GEO.

☒ Confirm that you have deposited or provided access to graph files (e.g. BED files) for the called peaks.

Data access links *May remain private before publication.*	All datasets are available in GEO under the accession number GSE255897 (https://www.ncbi.nlm.nih.gov/geo/query/acc.cgi?acc=GSE255897).
Files in database submission	Too many files to list here. Please check the GEO link.

Genome browser session (e.g. UCSC)	No longer applicable.

Methodology

Replicates	All ChIP-seq or other profiling experiment were performed on two biological replicates. The biological replicates were two independent differentiation experiments.
Sequencing depth	The ChIP-seq were paired-end 150bp. Sequencing depth and other metrics were provided in Supplemental Tables.
Antibodies	anti-CTCF (Active Motif, # 61311)
Peak calling parameters	Sample command: macs2 callpeak -t MUT.R1_sorted.bam MUT.R2_sorted.bam -c MUT_input.R1_sorted.bam -f BAM --outdir ./ -n MUT_CTCF --nomodel --verbose 3 -g mm -p 0.01 -m 5 50 1> MUT_CTCF.txt 2> MUT_CTCF.log&<br>ChIP-seq peaks were called by MACS2 for each biological replicate sample and pooled sample, using the pooled sample as control.
Data quality	Sufficient sequence depth were achieved according to ENCODE standards, each replicate has about 30 million usable fragments. Number of called peaks were compared to published data and it's quite comparable.
Software	Softwares include Bowtie2, samtools, MACS2, bedtools. Software versions were stated earlier.

Flow Cytometry

Plots

Confirm that:

☒ The axis labels state the marker and fluorochrome used (e.g. CD4-FITC).

☒ The axis scales are clearly visible. Include numbers along axes only for bottom left plot of group (a 'group' is an analysis of identical markers).

☒ All plots are contour plots with outliers or pseudocolor plots.

☒ A numerical value for number of cells or percentage (with statistics) is provided.

Methodology

Sample preparation	Single-cell suspensions were prepared and were fixed. At least 10^5 cells per experiment
Instrument	CytoFLEX
Software	The results were analyzed using FlowJo software.
Cell population abundance	At least 10^5 cells per experiment
Gating strategy	Wild-type mESC cells were used as a negative control.

☒ Tick this box to confirm that a figure exemplifying the gating strategy is provided in the Supplementary Information.
